# Positive Emotion Dysregulation in Opioid Use Disorder and Normalization by Mindfulness-Oriented Recovery Enhancement: A Secondary Analysis of a Randomized Clinical Trial

**DOI:** 10.1001/jamapsychiatry.2025.0569

**Published:** 2025-04-30

**Authors:** Eric L. Garland, Justin Hudak, Adam W. Hanley, Edward Bernat, Brett Froeliger

**Affiliations:** 1 Department of Psychiatry, University of California, San Diego, La Jolla; 2 Sanford Institute for Empathy and Compassion, University of California, San Diego, La Jolla; 3 College of Social Work, University of Utah, Salt Lake City; 4 College of Nursing, Florida State University, Tallahassee; 5 Department of Psychology, University of Maryland, College Park; 6 Departments of Psychiatry and Cognitive Neuroscience Systems, University of Missouri, Columbia

## Abstract

**Importance:**

It remains unknown whether difficulties in positive emotion regulation (ER) drive opioid use disorder (OUD) and whether these difficulties may be remediated.

**Objective:**

To test whether OUD is associated with attenuated positive ER and whether such positive emotion dysregulation may be treated by mindfulness-based intervention.

**Design, Setting, and Participants:**

In this mechanistic substudy of a randomized clinical trial, patients with chronic pain who did and did not meet criteria for OUD (OUD+ and OUD-, respectively) were recruited from primary care and pain clinics in Utah from January 2016 to and January 2020 and completed a positive ER task and questionnaires. A subsample of participants at risk for opioid misuse were randomized 1:1 to 8 weeks of Mindfulness-Oriented Recovery Enhancement (MORE) or supportive group (SG) therapy and then completed the ER task at posttreatment and questionnaires through 3-month follow-up. Data were analyzed from December 2022 to June 2024.

**Main Outcomes and Measures:**

The primary mechanistic outcome was parietal late positive potential (LPP) and P300 amplitude during positive ER. Secondary outcomes included self-reported anhedonia, positive affect, attention to positive information, and opioid craving.

**Results:**

The 160 participants used opioids for a mean (SD) duration of 9.7 (8.4) years, and 98 participants (61.3%) were classified as OUD+. Mean (SD) participant age was 53.7 (11.9) years, and 102 participants (63.8%) were female. A significant group × task strategy (View, Regulate) interaction was observed during positive ER, characterized by the OUD+ group exhibiting lower LPP responses during Regulate compared to View trials relative to the OUD- group (B = 1.91; 95% CI, 0.85-2.96; *P* < .001). A significant group × strategy interaction was also observed for the P300 (B = 1.40; 95% CI, 0.14-2.67; *P* = .03). LPP blunting during positive ER mediated the association between OUD status and elevated opioid craving. In the treatment subsample (n = 62), patients treated with MORE showed greater increases in the LPP during Regulate compared with View trials than patients treated with SG (B = 1.53; 95% CI, 0.33-2.73; *P* = .01). Additionally, MORE improved attention to positive information (B = 1.32; 95% CI, 1.14-5.57; *P* = .004), positive affect (B = 4.73; 95% CI, 1.22-8.24; *P* = .01), and anhedonia (B = 2.94; 95% CI, 0.58-5.31; *P* = .02) while reducing craving (B = −10.88; 95% CI, −21.29 to −0.48; *P* = .04) to a greater extent than SG. Higher positive ER efficacy predicted lower craving at follow-up (B = −2.38; 95% CI, −4.55 to −0.21; *P* = .03).

**Conclusions and Relevance:**

OUD among patients with chronic pain is characterized by difficulties in positive ER, evident in neurophysiological markers of salience detection and emotional elaboration. In this study, training in mindfulness and savoring via MORE enhanced positive ER, which in turn reduced craving, indicating that interventions that normalize reward system function may remediate OUD.

**Trial Registration:**

ClinicalTrials.gov Identifier: NCT02602535

## Key Points

**Question:**

Do people with opioid use disorder (OUD) and chronic pain exhibit positive emotion dysregulation, and can this dysregulation be remediated with treatment?

**Findings:**

In this mechanistic substudy of a randomized clinical trial, patients with chronic pain and OUD exhibited significantly greater blunting of electroencephalogram (EEG) markers of salience detection and motivated attention during upregulation of responding to positive emotional cues. Treatment with Mindfulness-Oriented Recovery Enhancement normalized EEG indices of positive emotion dysregulation.

**Meaning:**

OUD reduces the ability to savor natural healthy pleasure, but this problem might be remediated by cognitive interventions that include training in mindfulness and savoring.

## Teaser

This study tests whether opioid use disorder (OUD) is associated with attenuated positive emotion regulation and whether such positive emotion dysregulation may be treated by mindfulness-based intervention.

## Introduction

The US remains in an opioid crisis. In 2023, 8.9 million individuals in the US misused opioids, and 5.7 million had an opioid use disorder (OUD).^1^ The opioid crisis emerged in part from misuse of opioids.^2^ Although opioids relieve acute pain, their efficacy for chronic pain is variable.^3^ Protracted, high-dose opioid use may confer OUD risk due to the neuropsychopharmacologic effects of opioids on brain systems integral to reward processing, motivation, and emotion regulation.^4^

Allostatic models of the downward spiral linking chronic pain and OUD^5,6,7^ posit that prolonged opioid use in the context of pain and distress shifts hedonic set points in corticolimbic and corticostriatal circuitry mediating reward and thereby impairs proactive regulation of emotions. This allostatic process is thought to decrease natural reward responsivity and worsen dysphoria, compelling opioid use as a means of preserving hedonic equilibrium. Yet, chronic, compulsive opioid use further dysregulates hedonic function, resulting in a cycle of insensitivity to natural reward, emotion dysregulation, and drug seeking theorized to lead to OUD.^5^

Emotional responses and their regulation can be indexed by parietal electroencephalogram (EEG) event-related potentials (ERPs) (eg, late positive potential [LPP] and P300), which are known to reflect the processing of motivationally salient stimuli.^8^ The P300 is thought to index attentional deployment to an emotionally salient stimulus,^9,10^ whereas the LPP reflects motivation to sustain attention on the emotional features of^11,12^ and arousal in response to^13^ emotional stimuli. Changes in LPP^14,15,16^ and P300^17,18^ amplitude indicate emotion regulation efficacy.

Previous research has revealed associations between opioid misuse and decreased capacity to downregulate negative emotions through reappraisal as indexed by the LPP.^19^ However, in addition to problems with negative emotion regulation, addictive use of opioids is theorized to impair the ability to proactively upregulate responses to positive emotional stimuli (ie, savoring the pleasant sensory features of a naturally rewarding stimulus and the positive emotions and pleasurable sensations occasioned by that stimulus).^20^ Although reduced electrocortical reactivity to positive emotional stimuli^21,22^ and anhedonia^23^ have been observed among patients with OUD, no study has used task-related ERPs to assess whether people with OUD exhibit difficulties in proactively upregulating responses to positive emotional stimuli and whether such changes in positive emotion regulation (ER) predict addictive behavior.

To address this gap, an EEG experiment was conducted involving people with long-term opioid use who completed a validated positive ER task.^24,25,26^ Because opioids modulate central nervous responses, EEG differences between opioid users and healthy controls might reflect the acute pharmacologic action of the drug.^21^ Yet, OUD may have a unique association with attenuated neurophysiological response during positive ER above and beyond opioid pharmacodynamics. To disentangle the impact of addiction from opioid pharmacology, ERPs were assessed among patients with chronic pain and long-term opioid use who did and did not meet criteria for OUD. As an additional aim, this study sought to determine if difficulties in positive ER might be normalized by treatment. Thus, a subset of study participants were randomized to Mindfulness-Oriented Recovery Enhancement (MORE), an intervention designed to enhance positive ER through mindfulness, reappraisal, and savoring skills, or to a supportive group (SG) psychotherapy control condition. MORE has demonstrated efficacy for chronic pain, opioid misuse, and OUD across 7 published randomized clinical trials (RCTs) to date.^27,28,29,30,31^

It was hypothesized that relative to patients with chronic pain without OUD, those with OUD would exhibit reduced parietal LPP and P300 during positive ER (hypothesis 1). An allostatic model was tested to determine if attenuated LPP during positive ER mediated the association between OUD and opioid craving (hypothesis 2). Finally, it was hypothesized that MORE would improve LPP indices of positive ER and enhance positive affective responses following treatment (hypothesis 3).

## Methods

### Participants and Procedures

**Figure 1.**
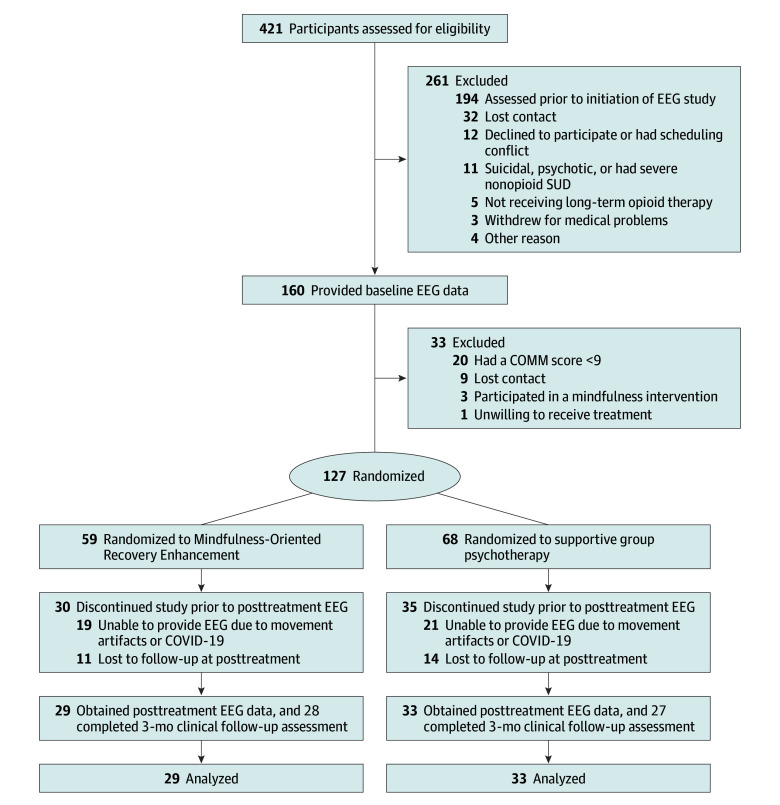
CONSORT Diagram Depicting the Study Flow COMM indicates Current Opioid Misuse Measure; EEG, electroencephalogram; SUD, substance use disorder.

Participants (N = 160) were recruited from primary care and pain clinics and met inclusion criteria if they reported chronic noncancer pain and had taken opioid analgesics for at least the past 90 days. Exclusion criteria were active suicidality or psychosis. For the mechanistic study reported in this manuscript, an EEG protocol was added to an RCT of opioid misuse interventions (NCT02602535) where EEG outcomes were not proposed as part of the original trial design. Primary trial outcomes were previously reported, following Consolidated Standards of Reporting Trials (CONSORT) reporting guidelines.^29^ Subsequent to the funding of the grant that supported this trial, the EEG protocol described herein was added as an ancillary mechanistic study overlaid on the RCT. As such, a subset of participants enrolled in the primary RCT provided EEG data for this mechanistic study. The trial protocol is available in Supplement 1, and the CONSORT flow diagram is depicted in Figure 1.

Participants gave written informed consent and completed a *DSM-5* assessment for OUD. Participants were classified into 1 of 2 diagnostic groups: those meeting criteria for OUD (OUD+ [n = 98]) or not meeting criteria (OUD- [n = 62]). Although they did not significantly differ on pain severity, OUD+ participants used higher opioid doses and had a shorter opioid use duration than OUD- participants (Table). Participants completed a training session to practice a positive ER task before performing the task while EEG was recorded. In addition, participants rated opioid craving (past week) on a 0 to 100 visual analog scale (VAS).^32^ The Positive and Negative Affect Schedule,^33^ the Attention to Positive and Negative Information Scale,^34^ the Snaith-Hamilton Anhedonia and Pleasure Scale,^35^ and the Current Opioid Misuse Measure (COMM)^36^ were also administered.

**Table.** Clinical and Sociodemographic Characteristics of the Opioid-Treated Chronic Pain Sample (N = 160) by Presence vs Absence of *DSM-5* Prescription Opioid Use Disorder (OUD+ and OUD-, Respectively)

Measure	No. (%)	
	OUD- (n = 62)	OUD+ (n = 98)
Age, mean (SD), y	57.9 (10.7)	51.0 (12.2)
Sex		
Female	38 (61.3)	64 (65.3)
Male	24 (38.7)	34 (34.7)
Race and ethnicity^a^		
African American	0	1 (1.0)
Hispanic/Latino	2 (3.2)	4 (4.1)
Native American	0	2 (2.0)
White	58 (93.5)	87 (88.8)
Other^b^	2 (3.2)	4 (4.1)
Estimated household income, $		
<25 000	10 (16.1)	37 (37.8)
25 000-49 000	21 (33.8)	25 (25.5)
50 000-99 999	20 (32.3)	24 (24.5)
≥100 000	11 (17.7)	11 (11.2)
Not reported	0	1 (1.0)
Primary pain location		
Low back	27 (58.7)	47 (55.9)
Joint or arthritis	8 (17.4)	8 (9.5)
Fibromyalgia	5 (10.9)	9 (10.7)
Headache	3 (6.5)	5 (6.0)
Neuropathic	3 (6.5)	8 (9.5)
Neck	0	2 (2.4)
Other	0	5 (6.0)
Average pain severity (range 0-10), mean (SD), BPI	5.3 (1.6)	5.1 (1.5)
Opioid type^c^		
Hydrocodone	24 (38.7)	23 (23.5)
Oxycodone	19 (30.6)	38 (38.8)
Tramadol	12 (19.4)	16 (16.3)
Morphine	7 (11.3)	13 (13.3)
Buprenorphine	3 (4.8)	7 (7.1)
Methadone	3 (4.8)	8 (8.2)
Fentanyl	3 (4.8)	6 (6.1)
Other	2 (3.2)	5 (5.1)
Duration of opioid use, mean (SD), y^d^	17.5 (10.7)	13.7 (11.0)
Morphine equivalent daily dose, mean (SD), mg^d^	72.1 (114.4)	127.4 (179.7)
OUD symptoms, mean (SD), No.^e^	0.4 (0.5)	3.9 (2.3)
Major depressive disorder diagnosis^f^	37 (59.7)	80 (81.6)
Nonopioid substance use disorder diagnosis^f^	0	11 (11.2)
Opioid craving VAS (range 0-100), mean (SD)^e^	15.9 (24.3)	32.6 (32.0)
Positive affect, mean (SD), PANAS score	27.6 (7.8)	29.9 (8.6)
Attention to positive information, mean (SD), APNIS score^g^	42.3 (6.2)	41.1 (6.8)
Anhedonia, mean (SD), SHAPS score^h^	24.4 (8.0)	25.6 (7.1)

Abbreviations: APNIS, Attention to Positive and Negative Information Scale; BPI, Brief Pain Inventory; PANAS, Positive and Negative Affect Schedule; SHAPS, Snaith-Hamilton Anhedonia and Pleasure Scale; VAS, visual analog scale.

^a^ Self-reported via demographic questionnaire.

^b^ Includes Asian participants, Pacific Islander participants, and those whose race or ethnicity was not disclosed.

^c^ Percentages do not sum to 100% because some participants reported using multiple opioid types.

^d^ *P* < .05.

^e^ *P* < .001.

^f^ *P* < .01.

^g^ Participants with severe OUD had significantly worse anhedonia than those with mild OUD (B = 4.49; 95% CI, 0.69-8.29; *P* = .02).

^h^ Participants with severe OUD had significantly worse attention to positive information than those with mild OUD (B = 4.96; 95% CI, 0.93-8.99; *P* = .01).

After completing this baseline pretreatment assessment, participants with COMM scores of 9 or higher (indicating opioid misuse risk; see eAppendix 2 in Supplement 2 for overlap between COMM and OUD groups) were randomized 1:1 to receive MORE or an SG treatment control condition. Following the 8-week interventions, they completed the positive ER task and clinical measures again. To understand how pre- to posttreatment changes in positive ER predicted proximal outcomes, clinical measures were also collected at 3-month follow-up. Patients with complete EEG data at pre- and posttreatment assessments (n = 62) were included in the treatment effects analysis of positive ER.

The protocol was approved by the University of Utah institutional review board (where the first author was faculty when the trial was conducted), and procedures complied with the Declaration of Helsinki. Participants were compensated $320 for completing these studies.

### Interventions

Participants were assigned to receive 8 weekly sessions of MORE or an SG led by a therapist (eAppendix 4 in Supplement 2). The empirically supported MORE intervention^27,28,29,30,31^ provided mindfulness training to cultivate meta-awareness and attentional control; reappraisal training to facilitate negative ER; and training in savoring pleasant events to enhance positive ER. Participants were instructed to use mindfulness to regulate opioid craving and to savor the pleasant sensory features of naturally rewarding objects and events, as well as the positive emotions and pleasurable sensations evoked by focusing on those objects or events. Participants were asked to engage in daily 15-minute mindfulness, reappraisal, and savoring practice sessions at home. The manualized active SG control condition in this study consisted of 8 weekly, 2-hour group sessions in which a Rogerian therapy approach was used to facilitate emotional expression and discussion of topics pertinent to pain and opioid misuse. SG participants were asked to engage at home in 15 minutes of journaling a day on pain and opioid-related themes.

### Positive ER Task

The validated positive ER task^25,37^ presented positive images in 2 blocked conditions: View Positive and Regulate Positive (eAppendix 3 in Supplement 2). On Regulate trials, to approximate savoring techniques and conform with typical “increase positive” instructions on ER tasks,^26^ participants were instructed to imagine experiencing the positive event occurring in the image and to focus on the pleasant aspects of the image while amplifying their own positive emotions and pleasant body sensations in response to the image. Participants practiced the View and Regulate strategies, provided affect ratings, and described their experience with each strategy to ensure task engagement.

### Electroencephalography

EEG data were continuously recorded using a 32-channel active sensor cap with Ag/AgCl electrodes (actiCap [Brain Products GmbH]). Additionally, electro-oculograms of vertical eye movements were recorded. All recordings were collected by an actiCHamp amplifier and BrainVision Recorder software (both Brain Products GmbH). For hypothesis testing, activity was assessed at Pz where the LPP and P300 are known to be maximal in affective picture viewing tasks.^38,39^ Independent component analysis was executed to semiautomatically remove ocular artifacts. Tasks were segmented and blocked by trial type for further processing. Considering the morphology of the observed ERP waveforms, conventions from previous studies were followed, which found the LPP maxima for evaluative positive ER strategies (eg, savoring) occurred between 600 and 1500 ms^40^; epochs were selected within this window for LPP analyses. P300 peaks were semiautomatically detected using an interval of 250 to 400 ms.

### Statistical Analysis

Power analyses were conducted in GLIMMPSE version 3.13. *P* values were 2-tailed. For hypothesis 1, based on previous studies, it was assumed ERP responses (eg, LPP) during the View and Regulate strategies would have an SD of 2.5 and that the correlation between View and Regulate ERPs (*r*) would be 0.5. For a power of 0.80 and a type I error rate of 0.05, 144 participants would be required to detect a moderate effect size OUD group × strategy interaction (ie, 1.25 uV in the LPP). For hypothesis 3, based on previous studies, we assumed an SD of 2.5 and a correlation between repeated ERPs over time of 0.65. Also based on previous studies, we assumed a correlation between View and Regulate ERPs of 0.5. For a power of 0.80 and a type I error rate of 0.05, 64 participants would be required to detect a large effect size of treatment (ie, 2.0-uV change in the LPP).

To test hypothesis 1, linear mixed models (LMM) were conducted to assess the group (OUD+ vs OUD-) × strategy (View vs Regulate) interaction on LPP and P300 to positive stimuli. LMM included random intercepts and were estimated with restricted maximum likelihood methods (REML) and Satterthwaite-approximated degrees of freedom. Exploratory 3-way interactions with epoch were included in the LMM, but given their nonsignificance (*P* values > .5), interactions with epoch are detailed in eTable 2 in Supplement 2. Given that opioid dose, pain severity, and age may influence EEG activity^41,42,43^ and that major depression and nonopioid substance use disorder diagnoses might influence reward responses, these potential confounders were controlled for in a sensitivity analysis. To test hypothesis 2, path analysis with bootstrapping (5000 samples)^44^ was performed in PROCESS version 2.16 in SPSS version 29.0 (IBM) to determine whether LPP activation during positive ER (Regulate − View difference score^25^) mediated the association between OUD diagnosis (OUD+ vs OUD-) and opioid craving. To test hypothesis 3, an LMM repeated measures analysis of covariance (ANCOVA) was used to examine the treatment (MORE vs SG) × strategy interaction on LPP during positive ER, adjusting for prerandomization differences in LPP during the View and Regulate conditions. In accordance with the classical ANCOVA approach for analyzing clinical trial outcomes,^45^ covarying prerandomization values performs statistical matching on the prerandomization scores and ensures that comparisons of postrandomization values by treatment group are independent of random baseline differences. Treatment effects on craving, attention to positive information, positive affect, and anhedonia outcomes (adjusted for prerandomization baseline values) were assessed with LMM repeated measures ANCOVA, using REML to handle missing data. The primary fixed effect of interest was the adjusted treatment main effect, which estimated the mean overall benefit of MORE vs SG through posttreatment and 3-month follow-up.

## Results

### Differences in Positive Emotion Regulation by OUD Diagnosis

**Figure 2.**
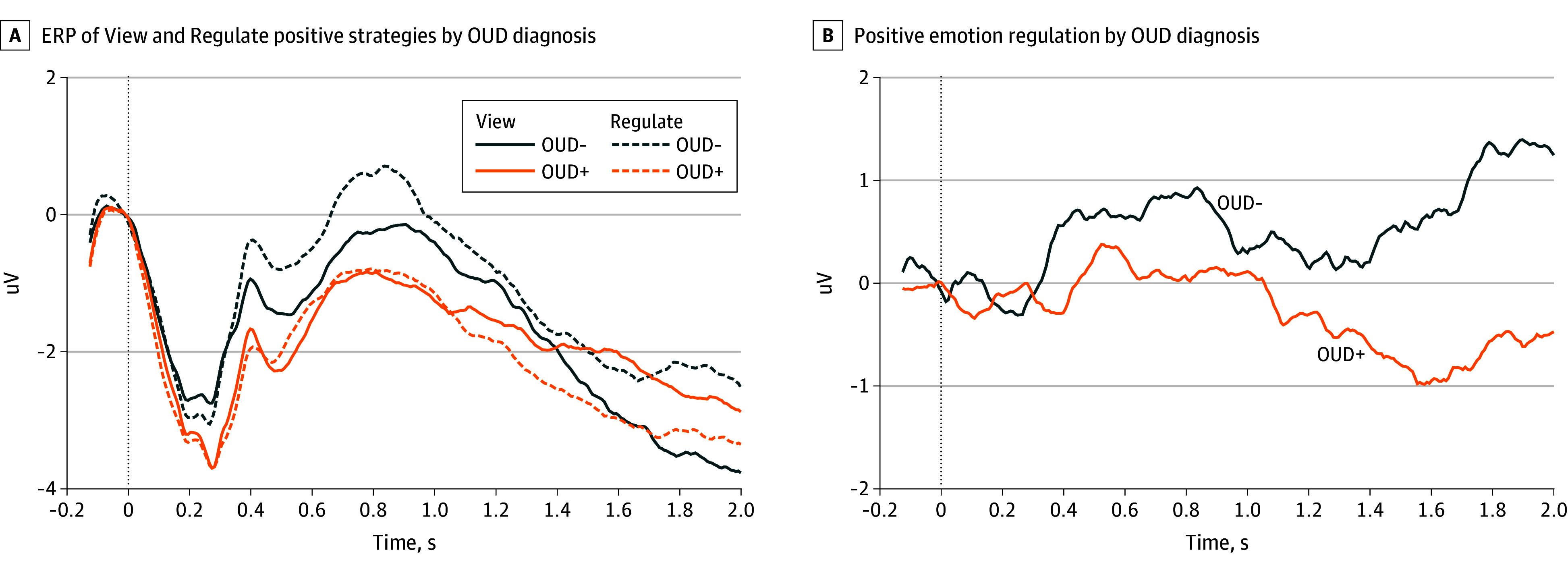
Event-Related Potential (ERP) of View Positive and Regulate Positive Strategies and Positive Emotion Regulation by Opioid Use Disorder (OUD) Diagnosis A, Grand mean ERP electroencephalogram waveforms (at Pz, in uV) for View Positive and Regulate Positive strategies by OUD diagnosis (OUD+ vs OUD-). B, Difference waves (at Pz, in uV) representing ERP changes during positive emotion regulation (Regulate − View) by OUD diagnosis.

**Figure 3.**
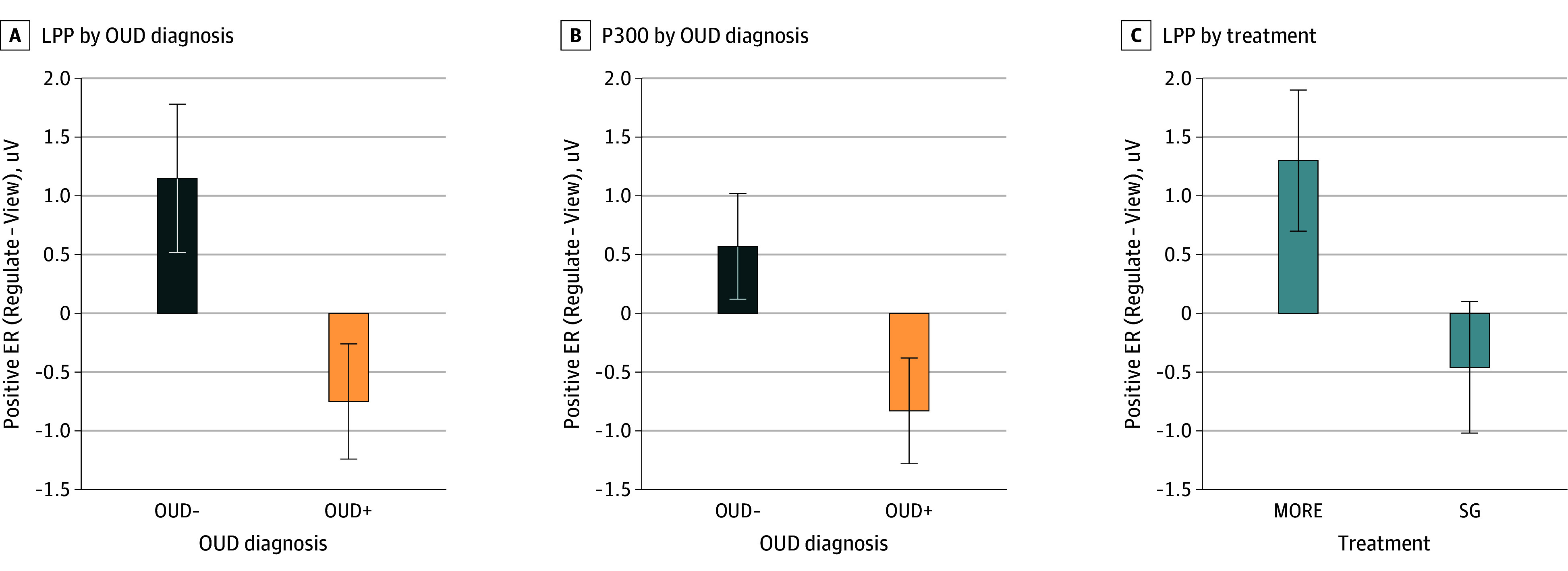
Event-Related Potential Changes from Pretreatment to Posttreatment With Mindfulness-Oriented Recovery Enhancement (MORE) and Supportive Group (SG) Therapy Positive emotion regulation is computed as the difference in amplitude at Pz between Regulate and View strategies (Regulate − View), in uV. Error bars represent 1 standard error. Compared with OUD- participants, OUD+ participants showed reduced late positive potential (LPP) (A) and P300 (B) during positive emotion regulation. C, Compared with participants in supportive group (SG) therapy, those treated with Mindfulness-Oriented Recovery Enhancement (MORE) showed increased LPP during positive emotion regulation. MORE vs SG treatment effects are expressed as estimated marginal means (standard error) of posttreatment LPP amplitude adjusted for pretreatment LPP amplitude during the View and Regulate conditions.

Mean (SD) participant age was 53.7 (11.9) years, and 102 participants (63.8%) were female. Analysis of LPP responses (Figure 2) revealed a significant group × strategy interaction (B = 1.91; 95% CI, 0.85-2.96; *P* < .001). Relative to OUD+ participants, OUD- participants evidenced significantly greater LPP responses on Regulate compared to View trials (Figure 3A). Neither the main effect of group (B = 1.04; 95% CI, −0.47 to 2.55; *P* = .18) nor the main effect of strategy (B = 0.19; 95% CI, −0.33 to 0.73; *P* = .46) were significant for the LPP.

Analysis of P300 responses (Figure 2) revealed a similar significant group × strategy interaction (B = 1.40; 95% CI, 0.14-2.67; *P* = .03). Relative to OUD+ participants, OUD- participants (Figure 3B) evidenced significantly greater P300 responses on Regulate compared to View trials. Neither the main effect of group (B = 0.97; 95% CI, −0.38 to 2.42; *P* = .19) nor the main effect of strategy (B = −0.13; 95% CI, −0.76 to 0.51; *P* = .69) were significant for the P300. The group × strategy interaction remained significant for the LPP and P300 in sensitivity analyses (eTables 2 and 3 in Supplement 2).

### LPP Mediation of Opioid Craving

Attenuated LPP activation (but not P300 activation) during upregulation of responding to naturally rewarding stimuli (Regulate − View) mediated the association between OUD status and opioid craving (B = 2.05; 95% CI, 0.23-4.65). Because this LPP positive ER index mediated craving, we then tested whether MORE could modulate the LPP during positive ER.

### Treatment Effects on Positive ER

**Figure 4.**
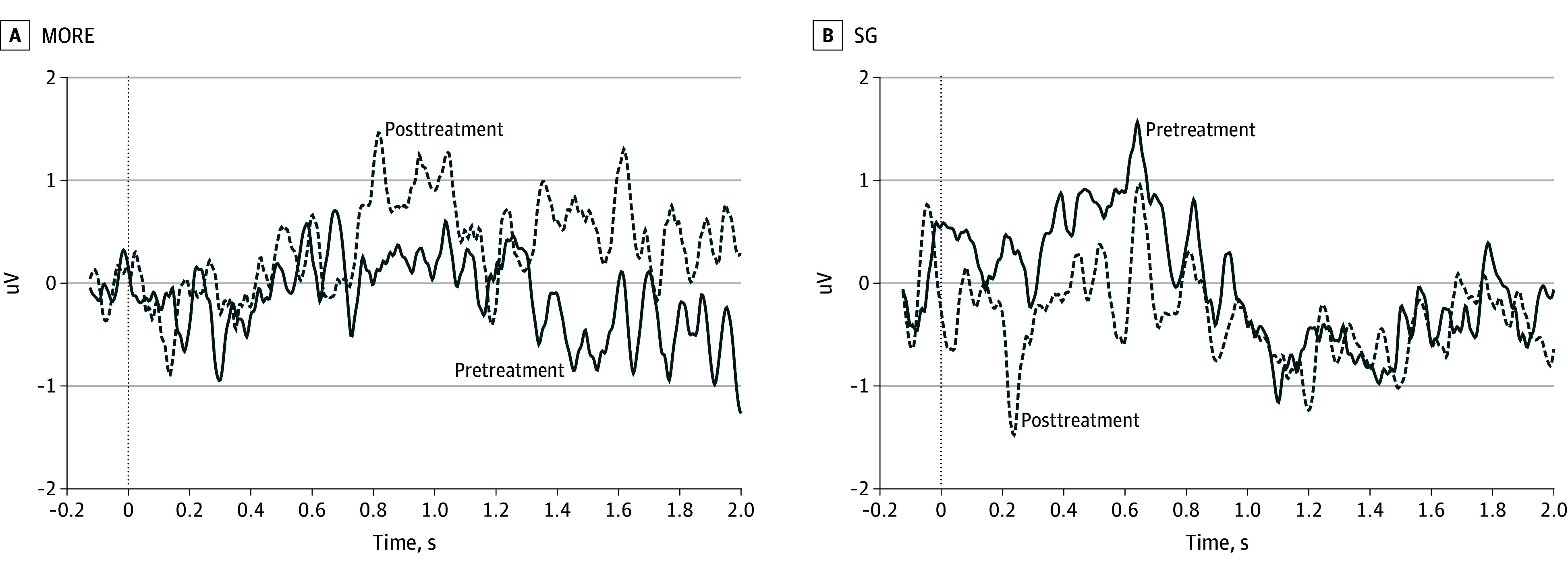
Effects of Opioid Use Disorder (OUD) Diagnosis and Treatment on Event-Related Potential Indices of Positive Emotion Regulation Difference waves (at Pz, in uV) representing event-related potential changes during positive emotion regulation (Regulate − View) from pre- to posttreatment with MORE and SG.

The treatment × strategy interaction was significant (B = 1.53; 95% CI, 0.33-2.73; *P* = .01) (see Figure 4 for difference waves and the eFigure in Supplement 3 for raw ERPs), indicating that patients treated with MORE showed greater LPP on Regulate compared with View trials than patients treated with SG (Figure 3C). Overall, 44.1% and 50.0% of participants in the MORE and SG treatment groups, respectively, were OUD+ at baseline; OUD status did not significantly moderate the impact of MORE on LPP responses.

Additionally, main effects of treatment on attention to positive information (B = 1.32; 95% CI, 1.14-5.57; *P* = .004), positive affect (B = 4.73; 95% CI, 1.22-8.24; *P* = .01), and anhedonia (B = 2.94; 95% CI, 0.58-5.31; *P* = .02) were significant, indicating that MORE resulted in greater improvements in these dimensions of positive affective responding compared to the SG treatment through the 3-month follow-up.

Importantly, MORE reduced opioid craving to a greater extent than the SG treatment (B = −10.88; 95% CI, −21.29 to −0.48; *P* = .04). Higher posttreatment positive ER efficacy (Regulate − View) indicated by the LPP predicted lower craving at 3-month follow-up (B = −2.38; 95% CI, −4.55 to −0.21; *P* = .03).

## Discussion

This study obtained evidence of positive emotion dysregulation among opioid-treated patients with chronic pain who met diagnostic criteria for OUD. Relative to those without OUD, patients with OUD exhibited decreased LPP and P300 during attempts to upregulate responding to stimuli representing natural rewards. Attenuated positive ER LPP amplitude mediated the association between OUD diagnosis and opioid craving. Further, treatment with MORE, a cognitive intervention that includes positive ER training, increased the LPP during proactive upregulation of responding to natural reward stimuli and improved various positive affective factors, suggesting that this evidence-based therapy might normalize positive ER in OUD.

According to the allostatic framework,^7,46^ the attempt to maintain hedonic homeostasis in the face of pain and distress via recurrent opioid use or misuse shifts brain reward set points, resulting in insensitivity to natural rewards. Inability to derive reward from healthful objects and events in the socioenvironment when coupled with loss of prefrontally mediated self-regulatory capacity is thought to compel opioid use as a means of preserving hedonic equilibrium, resulting in craving, opioid misuse, and, ultimately, OUD.^5^ Study findings provide support for this framework, with the timing of the observed neurophysiological attenuation suggesting that OUD-related positive emotion dysregulation occurs during salience detection (ie, P300), as well as during motivated attention and emotional elaboration (ie, LPP). Importantly, OUD diagnosis was associated with reduced neurophysiological indices of positive ER even after controlling for opioid dose, suggesting that opioid pharmacodynamics alone cannot explain the observed attenuation of positive ER capacity. Instead, these data suggest that enduring neurobiological changes occasioned by the addictive process might be responsible for emotion dysregulation.

The underlying neural generators of decreased positive ER could not be ascertained in the present study, yet prominent theoretical models posit that the ventral prefrontal cortex (PFC) modulates affective appraisal systems and striatal reward responses during proactive regulation of emotion.^47^ In that regard, illicit OUD is associated with reduced ventromedial PFC and caudate activity during savoring natural rewards relative to reappraising drug cues.^48^ Systems-level neuroscientific investigations of OUD-related positive emotion dysregulation that combine multimodal imaging techniques (eg, EEG and functional magnetic resonance imaging) are now indicated.

Neurophysiological evidence of impaired capacity to upregulate responsiveness to natural rewards reveals potential mechanistic treatment targets for future therapies aimed at ameliorating OUD. Although OUD+ participants exhibited attenuated neurophysiological indices of positive ER, positive affect and anhedonia did not significantly differ by OUD diagnosis. This dissociation between neural response and self-report (potentially indicative of a lack of insight) is consistent with other research demonstrating associations between OUD and lower metacognitive awareness, concomitant with task-related brain dysfunction.^49^ That said, when measured as continuous variables, OUD severity and opioid misuse scores were associated with positive affective decrements (eAppendix 8 in Supplement 2), and the opioid-using participants in this study (regardless of OUD diagnosis) reported significantly worse positive affect and anhedonia than healthy control samples.^33,35^ Here we showed that MORE, an empirically supported treatment^28^ that integrates mindfulness-based training in meta-awareness with systematic training in savoring natural rewards, may enhance positive affective processes and amplify neural indices of positive ER in people with OUD and/or opioid misuse. In prior RCTs, MORE decreased opioid misuse among people with chronic pain by 45%, more than doubling the effect of supportive therapy,^29^ and reduced relapse among people receiving medications for OUD.^31^ In the present study, increases in positive ER capacity following treatment were associated with decreased craving, consistent with previous pilot studies indicating that MORE’s craving-reducing effects are associated with increased psychophysiological indices of natural reward responsiveness.^50,51^ Whether MORE decreases addictive behaviors by durably restructuring brain reward processes^20^ supporting positive ER remains to be shown via replications in full-scale mechanistic RCTs. Ultimately, treatments that integrate evidence-based pharmacotherapies with behavioral interventions that normalize reward system function (eg, mindfulness, savoring, and compassion-based meditation) might produce robust and clinically significant improvements in OUD.

### Limitations

Although attenuated reward processing and emotion dysregulation may be diatheses that confer risk for developing OUD, it is reasonable to surmise that addictive use of opioids dysregulates neural circuitry instantiating hedonic functions. However, we found that LPP and P300 activation patterns were independent of opioid dose and duration. The present study design cannot disentangle acute and chronic effects of opioid exposure on positive ER. Similarly, the absence of a significant association with pain suggests that the observed positive ER effects are independent of pain experience and more strongly related to OUD. Because this study’s ER task did not include control images, it is unknown whether the observed emotion dysregulation is specific to positive stimuli or whether it extends to general ER difficulties, regardless of valence. Affect ratings on the ER task were also limited by reduced sensitivity and potential reporting biases. Additional longitudinal and experimental studies are needed to fully dismantle independent and interactive effects of chronic pain, opioid neuropsychopharmacology, and addictive behavior on hedonic regulation and to differentiate those factors from the endophenotypes that predispose patients with chronic pain to develop OUD.

Beyond the inability to determine whether the observed neurophysiological attenuation of positive ER was the cause, correlate, or consequence of OUD, the treatment effects analysis had sample size limitations. Although there was a substantial sample size for baseline EEG, comparatively fewer participants completed EEG at posttreatment due to the onset of the COVID-19 pandemic, which prevented psychophysiological data collection. Future large-scale trials are needed to replicate the observed effects of MORE on neural reward responses.

## Conclusions

Using an EEG biomarker of clinical target engagement with high temporal resolution, this study provides neurophysiological evidence of diminished positive ER capacity among patients with OUD and its potential normalization by MORE. Restructuring reward system function with mindfulness-based interventions and other integrative cognitive-affective training approaches may generate the learning signal required to restore adaptive hedonic regulation and thereby treat the pathophysiology undergirding opioid misuse and addiction.

## Notes

Supplement 1. Trial Protocol

Supplement 2. eAppendix 1. Study Flow eAppendix 2. Participants eAppendix 3. Assessment of Positive Emotion Regulation eAppendix 4. Study Interventions eAppendix 5. Power Analysis eAppendix 6. Expanded Statistical Analysis Details eTable 1. Main Effects and Interaction Effects for Hypothesis 1 eTable 2. Sensitivity Analyses for Hypothesis 1: LPP Sensitivity Analyses eTable 3. Sensitivity Analyses for Hypothesis 1: P300 Sensitivity Analyses eTable 4. Effects of Opioid Use Disorder (OUD) Status on the Late Positive Potential (LPP) and P300 Indices of Positive Emotion Regulation eTable 5. Effects of Treatment (MORE vs. SG) on the Late Positive Potential (LPP) Index of Positive Emotion Regulation eAppendix 7. Interactions Between Baseline OUD Diagnosis and Treatment Group eAppendix 8. Associations Among OUD Severity, Opioid Misuse, and Positive Affective Variables at Baseline eTable 6. Effects of Opioid Use Disorder (OUD) Status on Positive Affect Ratings During the ER Task eTable 7. Effects of Treatment on Positive Affect Ratings During the Task eFigure. Event-Related Potentials by Treatment Group at Pre- and Post-Treatment eTable 8. Treatment Effects on Self-Report Variables Through 3-Month Follow-Up eReferences.

Supplement 3. Data Sharing Statement
