# Supplementary material for: Positive Emotion Dysregulation in Opioid Use Disorder and Normalization by Mindfulness-Oriented Recovery Enhancement: A Secondary Analysis of a Randomized Clinical Trial
Source: JAMA Psychiatry. 2025 Apr 30;82(7):654–62. doi: 10.1001/jamapsychiatry.2025.0569 (PMC12044540; doi:10.1001/jamapsychiatry.2025.0569)
Supplement: Supplement 2. — eAppendix 1. Study Flow eAppendix 2. Participants eAppendix 3. Assessment of Positive Emotion Regulation eAppendix 4. Study Interventions eAppendix 5. Power Analysis eAppendix 6. Expanded Statistical Analysis Details eTable 1. Main Effects and Interaction Effects for Hypothesis 1 eTable 2. Sensitivity Analyses for Hypothesis 1: LPP Sensitivity Analyses eTable 3. Sensitivity Analyses for Hypothesis 1: P300 Sensitivity Analyses eTable 4. Effects of Opioid Use Disorder (OUD) Status on the Late Positive Potential (LPP) and P300 Indices of Positive Emotion Regulation eTable 5. Effects of Treatment (MORE vs. SG) on the Late Positive Potential (LPP) Index of Positive Emotion Regulation eAppendix 7. Interactions Between Baseline OUD Diagnosis and Treatment Group eAppendix 8. Associations Among OUD Severity, Opioid Misuse, and Positive Affective Variables at Baseline eTable 6. Effects of Opioid Use Disorder (OUD) Status on Positive Affect Ratings During the ER Task eTable 7. Effects of Treatment on Positive Affect Ratings During the Task eFigure. Event-Related Potentials by Treatment Group at Pre- and Post-Treatment eTable 8. Treatment Effects on Self-Report Variables Through 3-Month Follow-Up eReferences. [file jamapsychiatry-e250569-s002.pdf]

Supplemental Online Content

Garland EL, Hudak J, Hanley AW, Bernat E, Froeliger B. Positive emotion dysregulation in OUD and normalization by Mindfulness-Oriented Recovery Enhancement. *JAMA Psychiatry*. Published online April 30, 2025. doi:10.1001/jamapsychiatry.2025.0569

eAppendix 1. Study Flow

eAppendix 2. Participants

eAppendix 3. Assessment of Positive Emotion Regulation

eAppendix 4. Study Interventions

eAppendix 5. Power Analysis

eAppendix 6. Expanded Statistical Analysis Details

eTable 1. Main Effects and Interaction Effects for Hypothesis 1

eTable 2. Sensitivity Analyses for Hypothesis 1: LPP Sensitivity Analyses

eTable 3. Sensitivity Analyses for Hypothesis 1: P300 Sensitivity Analyses

eTable 4. Effects of Opioid Use Disorder (OUD) Status on the Late Positive Potential (LPP) and P300 Indices of Positive Emotion Regulation

eTable 5. Effects of Treatment (MORE vs. SG) on the Late Positive Potential (LPP) Index of Positive Emotion Regulation

eAppendix 7. Interactions Between Baseline OUD Diagnosis and Treatment Group

eAppendix 8. Associations Among OUD Severity, Opioid Misuse, and Positive Affective Variables at Baseline

eTable 6. Effects of Opioid Use Disorder (OUD) Status on Positive Affect Ratings During the ER Task

eTable 7. Effects of Treatment on Positive Affect Ratings During the Task

eFigure. Event-Related Potentials by Treatment Group at Pre- and Post-Treatment

eTable 8. Treatment Effects on Self-Report Variables Through 3-Month Follow-Up

eReferences.

This supplemental material has been provided by the authors to give readers additional information about their work.

© 2025 Garland EL et al. *JAMA Psychiatry*.

eAppendix 1. Study Flow

All participants first completed the baseline study (assessing the effects of OUD diagnosis on neurophysiological indices of positive ER, hypotheses 1 and 2). Data from this baseline study were also used as pre-treatment data for participants who then went on to participate in the randomized controlled trial (RCT) studying the effects of MORE on positive ER (hypothesis 3) at post-treatment. This baseline EEG and clinical assessment was completed within four weeks of starting the study interventions (Time 1). All 160 participants completed one EEG and clinical assessment at Time 1, and 62 participants also completed a second EEG and clinical assessment at post-treatment (Time 2, within four weeks after completing the study interventions). Of these participants, 57 completed an additional clinical assessment consisting of questionnaires at Time 3 (3-month follow-up).

© 2025 Garland EL et al. *JAMA Psychiatry*.

eAppendix 2. Participants

The baseline study included 160 participants with chronic pain and long-term opioid use. These participants were evaluated for OUD diagnosis and opioid misuse risk (via the Current Opioid Misuse Measure, COMM). Of this sample, 140 participants met criteria for opioid misuse risk on the COMM (COMM<9). The COMM is not a direct measure of opioid misuse, but rather ascertains opioid misuse risk across a broad range of signs and behaviors that extend beyond direct opioid misuse behaviors (e.g., opioid dose escalation, self-medication of negative affect with opioids, obtaining opioids from multiple physicians), including emotional volatility, relationship concerns, and cognitive impairments. These latter factors, while statistically predictive of actual opioid misuse, are not direct measures of opioid misuse. In our sample, COMM scores were strongly, though not perfectly, correlated with OUD severity (i.e., # of DSM-5 OUD symptoms), r=0.58, p<.001. The overlap between OUD diagnosis and opioid misuse risk status in our baseline sample is shown in the crosstab below:

Crosstab

Count

		COMM score of 9 or greater		
		0	1	Total
OUD Diagnosis at Baseline	0	14	48	62
	1	6	92	98
Total		20	140	160

Recent research using the NDSUH indicates many individuals with prescription OUD diagnosis do not report overt opioid misuse behaviors.[1] Although in the Han et al. study,[1] patients with moderate-to-severe OUD were more likely to endorse opioid misuse, OUD is not synonymous with opioid misuse.

Differences in OUD criteria by OUD diagnosis groups in our sample are shown in the table below.

DSM-5 OUD Criteria	OUD-	OUD+
1) Take opioids in larger amounts or over a longer period of time than you intended to.	3 (4.8%)	58 (59.2%)
2) Have a strong desire to cut down or control opioid use. Try unsuccessfully to cut down or control opioid use.	14 (22.6%)	74 (75.5%)
3) Spend a great deal of time trying to obtain opioids, use opioids, or recover from the effects of opioids.	0 (0.0%)	33 (33.7%)
4) Experience craving for opioids. Experience a strong desire or urge to take opioids.	6 (9.7%)	58 (59.2%)

© 2025 Garland EL et al. *JAMA Psychiatry*.

5) Fail to fulfill obligations at work, school, or home because of taking opioids.	2 (3.2%)	30 (30.6%)
6) Continue taking opioids despite having social or relationship problems caused by or made worse by the effects of opioids.	0 (0.0%)	36 (36.7%)
7) Give up or reduce important social, work, or recreational activities because of taking opioids.	0 (0.0%)	36 (36.7%)
8) Repeatedly take opioids in situations in which it was physically hazardous.	0 (0.0%)	25 (25.5%)
9) Continue taking opioids despite knowing of an ongoing physical or psychological problem that may have been caused by or made worse by taking opioids.	1 (1.6%)	38 (38.8%)
10) *Tolerance:* Experience a need for increased amounts of opioids to achieve the desired effect. Experience a diminished effect with continued use of the same amount of an opioid. ***	15 (24.2%)	68 (69.4%)
11) *Withdrawal:* Experience withdrawal symptoms as a result of not taking opioids. Took opioids to relieve or avoid withdrawal symptoms. ***	15 (24.2%)	62 (63.3%)

Routes of administration for opioids were similar across OUD diagnosis groups, with the overwhelming majority of subjects in both groups (93.8%) taking oral opioids (OUD groups did not differ on the proportion of subjects taking oral/non-oral opioids, $\chi^2=0.007$, p=0.93).

© 2025 Garland EL et al. *JAMA Psychiatry*.

eAppendix 3. Assessment of Positive Emotion Regulation

Emotion regulation task. The study utilized an emotion regulation paradigm[2,3] that was comprised of 60 trials, each presented for 6 seconds, separated by a 500 ms fixation cross. There were two conditions, each presented in one of two randomized, counterbalanced blocks: "view positive," and "regulate positive." Participants were instructed to View or Regulate responses to natural reward stimuli. On View trials, participants were instructed to simply attend to images of naturally rewarding stimuli (e.g., social affiliation, natural beauty, athletic victories, etc.) validated in prior studies.[4] On Regulate trials, to approximate mindful savoring techniques[5] and conform with typical "increase positive" instructions on emotion regulation tasks,[6] participants were instructed to imagine experiencing the positive event occurring in the image, and to focus on the pleasant aspects of the image, appreciate the pleasant body sensations occasioned by the image, and amplify their own positive emotional response to the image. Participants rated positive affect on a 5-button response box (1-5) once after the "view positive" and once after the "regulate positive" blocks. In a training session prior to psychophysiological assessment, participants practiced the view and regulatory strategies, provided affect ratings, and described their experience of the positive affective stimuli and each strategy to a trained research assistant to ensure comprehension of the instructions. Psychophysiological assessment did not commence until participants demonstrated comprehension of each task condition and could accurately implement the appropriate strategy corresponding to each condition (i.e., view vs regulate).

EEG. To control for acute withdrawal effects on EEG responses, on the day of the EEG session, patients were instructed to take their opioids as they normally would at their typical dose (e.g., hydrocodone PO q4-6hr PRN). Routes of opioid administration were similar across groups, with the overwhelming majority of subjects in both groups (93.8%) taking oral opioids (OUD groups did not differ on the proportion of subjects taking oral/non-oral opioids, $\chi^2=0.007$, $p=0.93$). All participants were experiencing acute effects of their opioid medication during the 1 hour EEG session, and were not in acute withdrawal. EEG was continuously recorded from 10 midline scalp sites (Fz, F3, F4, FC1, FC2, FCz, Cz, CP1, CP2, PZ) using a 32-channel active sensor cap with Ag/AgCl electrodes (actiCap GmbH, Herrsching, Germany). All recordings were collected by an actiCHamp amplifier (Brain Products GmbH, Gilching, Germany). Data were acquired at a sampling rate of 500 Hz, a resolution of 0.489 μV and an amplification cutoff of 140 Hz, with impedances kept below 10kΩ.

EEG data preprocessing was conducted using a custom MATLAB[7] script set developed by the authors, containing both original and EEGLAB[8] functions, while ERP analysis was performed in the Psychophysiological Toolbox.[9] A low-pass filter of 50hz was applied to the continuous data, and then ERP epochs were created beginning 500ms pre-stimulus and ending 2000ms post-stimulus. Independent component analysis was executed to semi-automatically remove ocular artifacts from EEG recordings. Tasks were segmented and then blocked by trial type for further processing. For each individual, epochs were ranked according to number of extreme (> ±150mV) data points across all channels, and the worst 5% of epochs were removed. Additionally, individual channels were interpolated across all data if they exceeded the threshold of 5 standard deviations in the domains of kurtosis and activity probability. After baseline (500 to 100-ms pre-stimulus) correction occurred, each epoch was evaluated separately and channels with extreme (> ±150mV) data points were interpolated only for that epoch, while epochs with more than 2 bad channels were rejected and removed from the data. A final visual inspection was conducted to remove epochs with unusual artifacts. ERP component scores were extracted and exported for further statistical analyses. Considering the morphology of the observed ERP waveforms, we followed conventions from previous studies which found the LPP maxima for evaluative positive ER strategies (e.g., savoring) to occur

© 2025 Garland EL et al. *JAMA Psychiatry*.

between 600-1500 ms;[10] we defined the LPP ROI as average voltage across two successive time windows: 600-1000 ms and 1000-1500 ms. P300 peaks were semi-automatically detected using an interval of 250-400ms. For hypothesis testing, we assessed activity at Pz where the LPP and P300 were maximal, consistent with previous literature.[11,12]

© 2025 Garland EL et al. *JAMA Psychiatry*.

eAppendix 4. Study Interventions

MORE sessions followed a treatment manual.[13] MORE sessions focused on applying mindfulness, reappraisal, and savoring skills to promote positive psychological health, produce analgesia, and reduce maladaptive affective and appetitive responses. In MORE, participants were taught foundational mindfulness skills to promote self-awareness, self-regulation, and self-transcendence. As the MORE treatment sequence progressed, mindfulness training synergized reappraisal and savoring techniques not found in other mindfulness-based interventions. To cope with symptoms of pain and craving, participants were first taught unique mindful breathing and body scan meditations designed to decompose experiences of pain and craving into their constituent sensations (e.g., heat, tightness, tingling, vibration), as well as to increase awareness of the center, edges, and permeability (versus solidity) of these sensations, and any adjacent or distal pleasant sensations. Next, participants were taught reappraisal techniques, in which integrated mindfulness and cognitive restructuring skills were used to disengage from negative appraisals and generate adaptive reappraisals to reduce distress and opioid misuse. Finally, participants were taught savoring skills in which they directed mindful attention towards the pleasant sensory features of naturally rewarding objects and events while cultivating meta-awareness of the positive emotions and pleasurable sensations occasioned by rewarding life experiences. That is, in Session 4, participants practiced a mindful savoring technique in which they were first instructed to mindfully attend to the pleasant colors, textures, and scents of a rose, as well as the touch of its petals against the skin, while remaining sensitive to their own emotional response to the flower. When participants become aware of sensations of pleasure or positive emotions, they are instructed to turn attention inward, and mindfully savor the pleasant internal experience and the arising of any higher-order affective associations until they begin to fade, at which point attention shifts outward again to appreciate the flower once more. Hypothetically, this toggling of exteroceptive and interoceptive attention on pleasant perceptions, sensations, cognitions, and emotions may overcome the “hedonic treadmill effect” to intensify and prolong the pleasant experience. After learning this technique, participants are instructed to practice mindful savoring with other, more personally meaningful pleasant stimuli that naturally occur their everyday lives. Psychoeducation content of the MORE session topics included the following: 1) discriminating between nociception, pain, and suffering; 2) gaining awareness of automaticity in chronic pain and opioid use; 3) disrupting the link between negative emotions, catastrophizing, and pain experience through reappraisal; 4) savoring pleasant experiences to remediate reward dysregulation; 5) regulating opioid craving through mindful awareness and mindful reappraisal; 6) preventing opioid misuse by disrupting the link between stress and craving; 7) cultivating self-transcendence and meaning in life by connecting with something greater than the self; and 8) developing a mindful recovery plan. Participants were instructed to engage in 15 minutes of mindfulness, reappraisal, and savoring practice a day.

The supportive psychotherapy intervention involved discussion of topics pertinent to chronic pain and opioid misuse that were selected to roughly match corresponding themes in the MORE intervention, including: physical and psychological dimensions of pain; stress and coping; stigma of opioid use and misuse; use of opioids to alleviate negative emotions; and opioid-related adverse effects. During these supportive psychotherapy discussions, no cognitive-behavioral or mindfulness and acceptance-based skills were discussed or taught. Instead, supportive psychotherapy participants were guided via client-centered reflective listening techniques to disclose feelings and thoughts about group topics, as well as to provide advice and emotional support for their peers. During the supportive psychotherapy intervention, therapists engaged in an array of general therapeutic behaviors, including building rapport, presenting unconditional positive regard, active listening, empathic responding, elicitation of

emotional expression, and promoting mutual support between group members. No specific therapeutic skill training (e.g. mindfulness training) was provided. This control intervention, which typifies a widely-available form of conventional, process-oriented group therapy, was found in three prior RCTs to have equivalent perceived credibility (via the Treatment Credibility Questionnaire, Borkovec & Nau, 1972) to mindfulness-based interventions, including MORE (Garland et al., 2010, 2014; Gaylord et al., 2011).

© 2025 Garland EL et al. *JAMA Psychiatry*.

eAppendix 5. Power Analysis

We conducted a power analysis in GLIMMPSE (General Linear Mixed Model Power and Sample Size) to determine the sample size needed for a linear mixed model (a repeated measures test using the Hotelling-Lawley Trace) to test for an OUD Group by Strategy (View, Regulate) interaction on ERPs (e.g., LPP). Based on previous studies, we predicted ERP responses (e.g, LPP) during the View and Regulate strategies would have a standard deviation of 2.5. Also based on previous studies, we expected the correlation between ERPs under the View and Regulate conditions would be 0.5. For a desired power of 0.80 and a Type I error rate of 0.05, 144 participants would be required to detect a moderate effect size Group X Strategy interaction on ERPs (i.e., 1.25 uV in the LPP).

We conducted another power analysis in GLIMMPSE to determine the sample size needed for a linear mixed model (a repeated measured test using the Hotelling-Lawley Trace test) to test for a Treatment (MORE, SG) by Strategy (View, Regulate) interaction over time on the LPP. Based on previous studies, the standard deviation was assumed to be 2.5, and the correlation between repeated LPPs over time was assumed to be 0.65. Also based on previous studies, we expected the correlation between ERPs under the View and Regulate conditions would be 0.5. For a desired power of 0.80 and a Type I error rate of 0.05, 64 participants would be required to detect a large effect size of treatment on ERP (i.e., 2.0 uV change in the LPP).

eAppendix 6. Expanded Statistical Analysis Details

To test hypothesis 1, we conducted linear mixed models (LMM) to assess the Group (OUD+ vs OUD-) X Strategy (View vs. Regulate) interaction on baseline-corrected LPP and P300 to positive stimuli. LMM included random intercepts for participant and were estimated with restricted maximum likelihood methods (REML) with Satterthwaite-approximated degrees of freedom. Given that opioid dose, opioid duration, pain severity, and age may differ between OUD+ and OUD- groups and have been shown to influence EEG activity,[14–16] we controlled for these variables in a sensitivity analysis. Given their potential effects on reward responses, we also controlled for major depression and non-opioid SUD diagnoses in the sensitivity analysis. To test hypothesis 2, path analyses with bootstrapping (5000 samples)[17] were performed in PROCESS 2.16 software to determine whether LPP activation during proactive regulation of responding to naturally rewarding stimuli (Regulate – View difference score[18]) mediated the association between OUD status (OUD+ versus OUD-) and opioid craving. We employed robust estimation techniques (i.e., bootstrapping with 5000 bootstrap samples) to estimate the regression coefficients and robust standard errors for the mediation model testing hypothesis 2. We employed a heteroscedasticity consistent standard error and covariance matrix estimator (HC0 Huber-White) that is robust to potential non-normality in the data.

To test hypothesis 3, we used a LMM repeated-measures ANCOVA to examine the Treatment (MORE vs SG) X Strategy (View vs. Regulate) on this baseline-corrected LPP window during positive ER, adjusted for pre-randomization differences in LPP during the View and Regulate conditions. In accordance with the classical ANCOVA approach endorsed by Frison and Pocock for analyzing clinical trial outcomes,[19] covarying pre-randomization values performs statistical matching on the pre-randomization scores and ensures that comparisons of post-randomization values by treatment group are independent of random baseline differences. The effect of Treatment on opioid craving, attention to positive information, positive affect, and anhedonia outcomes (adjusted on pre-randomization baseline values) through post-treatment and 3-month follow-up was assessed with LMM repeated-measures ANCOVA, using REML to handle missing data. The primary fixed effect of interest was the adjusted treatment main effect, which estimated the mean overall benefit of MORE vs SG across both follow-up visits. Although not a primary *a priori* hypothesis, in LMMs testing effects of OUD Diagnosis (OUD+ vs. OUD-) and Treatment (MORE vs. SG), we also included an epoch factor to test whether the effects varied across earlier (600-1000ms) and later LPP components (1000-1500ms). The three-way interactions with epoch were non-significant ($p>0.50$).

© 2025 Garland EL et al. *JAMA Psychiatry*.

eTable 1. Main Effects and Interaction Effects for Hypothesis 1

LPP	Estimate (SE)	p-value
Group	1.04 (0.77)	0.18
Strategy	0.19 (0.27)	0.46
Group X Strategy	1.91 (0.54)	<.001
P300		
Group	0.97 (0.74)	0.19
Strategy	-0.13 (0.32)	0.69
Group X Strategy	1.40 (0.64)	0.03

© 2025 Garland EL et al. *JAMA Psychiatry*.

eTable 2. Sensitivity Analyses for Hypothesis 1: LPP Sensitivity Analyses

Fixed Effects Omnibus Tests

	F	df	df (res)	p
**OUD Diagnosis**	0.613	1	146.000	0.435
**Strategy (Regulate vs. View)**	0.073	1	456.000	0.787
**Epoch**	25.798	1	456.000	<.001
**Age**	0.322	1	146.000	0.572
**Pain Severity**	0.344	1	146.000	0.559
**Opioid Dose (MME)**	0.282	1	146.000	0.596
**Opioid Duration**	0.785	1	146.000	0.377
**SUD Diagnosis**	0.667	1	146.000	0.415
**MDD Diagnosis**	1.594	1	146.000	0.209
**OUD Diagnosis X Strategy**	9.467	1	456.000	0.002
**OUD Diagnosis X Epoch**	2.721	1	456.000	0.100
**Regulate X Epoch**	0.002	1	456.000	0.962
**OUD Diagnosis X Regulate X Epoch**	0.029	1	456.000	0.864

Our key hypothesis was identified in gray.

© 2025 Garland EL et al. *JAMA Psychiatry*.

eTable 3. Sensitivity Analyses for Hypothesis 1: P300 Sensitivity Analyses

Fixed Effects Omnibus Tests

	F	df	df (res)	p
**OUD Diagnosis**	0.009	1	146.000	0.925
**Strategy (Regulate vs. View)**	0.795	1	152.000	0.374
**Age**	1.035	1	146.000	0.311
**Pain Severity**	0.551	1	146.000	0.459
**Opioid Dose (MME)**	0.004	1	146.000	0.949
**Opioid Duration**	0.197	1	146.000	0.658
**MDD Diagnosis**	3.639	1	146.000	0.058
**SUD Diagnosis**	0.618	1	146.000	0.433
**OUD Diagnosis X Strategy**	4.373	1	152.000	0.038

Our key hypothesis was identified in gray.

© 2025 Garland EL et al. *JAMA Psychiatry*.

eTable 4. Effects of Opioid Use Disorder (OUD) Status on the Late Positive Potential (LPP) and P300 Indices of Positive Emotion Regulation

	OUD-<br>mean (SE)	OUD+<br>mean (SE)	Between-groups test
LPP	1.15 (0.63)	-0.75 (0.49)	*
P300	0.57 (0.50)	-0.83 (0.40)	*

Positive emotion regulation is computed as the difference in LPP/P300 during Regulate - View strategies. OUD effects are expressed as estimated marginal means (standard error) from linear models. * $p<0.05$

© 2025 Garland EL et al. *JAMA Psychiatry*.

eTable 5. Effects of Treatment (MORE vs. SG) on the Late Positive Potential (LPP) Index of Positive Emotion Regulation

	MORE mean (SE)	SG mean (SE)	Between-groups test
Treatment Effect (LPP)	1.30 (0.60)	-0.46 (0.56)	*

Positive emotion regulation is computed as the difference in LPP during Regulate - View strategies. MORE vs. SG treatment effects are expressed as estimated marginal means (standard error) of post-treatment LPP amplitude from linear models adjusted for pre-treatment LPP amplitude during the view and regulate conditions. * $p<0.05$

© 2025 Garland EL et al. *JAMA Psychiatry*.

eAppendix 7. Interactions Between Baseline OUD Diagnosis and Treatment Group

In a separate analysis, we included OUD status as a factor in a LMM. The two-way interaction between treatment group and OUD diagnosis (B=0.92, SE=1.59, p=0.56) and the three-way treatment X OUD diagnosis X strategy interactions were non-significant (B=1.39, SE=1.32, p=0.29), indicating that the effect of MORE did not differ significantly by baseline OUD status, suggesting that both groups benefitted similarly in terms of ameliorating of their LPP response by MORE. Thus, OUD diagnosis was removed from treatment effects analysis models.

© 2025 Garland EL et al. *JAMA Psychiatry*.

eAppendix 8. Associations Among OUD Severity, Opioid Misuse, and Positive Affective Variables at Baseline

When OUD was measured as a binary (OUD+ vs. OUD-) variable, OUD groups did not differ in self-reported measures of positive affect and anhedonia, yet when measured as continuous variables, OUD severity (measured continuously as the number of DSM-5 OUD symptoms, r =-0.19, p=0.02) and COMM opioid misuse scores (r = -0.20, p = 0.01) were significantly associated with lower positive affect. Also, COMM opioid misuse scores were significantly associated with higher anhedonia (r= 0.22, p=0.007).

When OUD severity was measured as a 3-level categorical variable (mild, moderate, and severe OUD), OUD severity groups significantly differed on anhedonia ($F_{2,92}$= 4.17, p=0.019) and attention to positive information ($F_{2,92}$= 4.55, p=0.013), but not on positive affect scores ($F_{2,92}$= 2.36, p=0.10). That is, in Bonferroni-corrected posthoc tests, participants with severe OUD had significantly worse anhedonia (p=0.015) and attention to positive information (p=0.010) than those with mild OUD.

© 2025 Garland EL et al. *JAMA Psychiatry*.

eTable 6. Effects of Opioid Use Disorder (OUD) Status on Positive Affect Ratings During the ER Task

OUD Status	Strategy	Mean	Std. Error	95% Confidence Interval Lower Bound	95% Confidence Interval Upper Bound
OUD-	View	3.914	.116	3.685	4.143
	Regulate	3.931	.134	3.666	4.196
OUD+	View	3.847	.096	3.658	4.036
	Regulate	3.882	.111	3.664	4.101

Group p=0.69; Strategy p=0.72; Group X Strategy p=0.90

© 2025 Garland EL et al. *JAMA Psychiatry*.

eTable 7. Effects of Treatment on Positive Affect Ratings During the Task

Treatment	Time	factor	Mean	Std. Error	95% Confidence Interval Lower Bound	Upper Bound
SG	Pre	View	3.500	.205	3.089	3.911
		Regulate	3.406	.249	2.908	3.904
	Post	View	3.000	.262	2.476	3.524
		Regulate	3.094	.278	2.537	3.650
MORE	Pre	View	3.179	.219	2.740	3.618
		Regulate	3.250	.266	2.717	3.783
	Post	View	3.250	.280	2.690	3.810
		Regulate	3.429	.297	2.833	4.024

For parallelism with the LPP treatment analysis, ANCOVA was performed, examining the Group X Strategy interaction, adjusting for pre-treatment positive affect. Group p=0.48; Strategy p=0.95; Group X Strategy p=0.71

© 2025 Garland EL et al. *JAMA Psychiatry*.

eFigure. Event-Related Potentials by Treatment Group at Pre- and Post-Treatment

After adjusting for pre-randomization differences in LPP during the View and Regulate conditions, the Treatment X Strategy interaction on the LPP was statistically significant (B=1.53, SE=0.61, p=0.013) indicating that MORE resulted in greater increases during the regulate strategy (e.g., savoring) relative to the view strategy compared to supportive group psychotherapy.

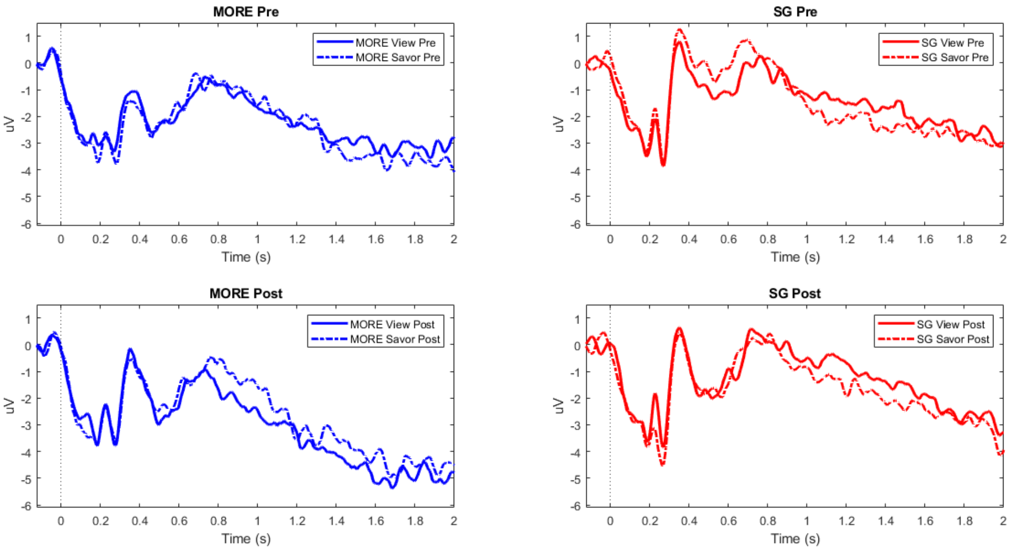

© 2025 Garland EL et al. *JAMA Psychiatry*.

eTable 8. Treatment Effects on Self-Report Variables Through 3-Month Follow-Up

	MORE mean (SE)	SG mean (SE)	p-value
Attention to Positive Information (APNIS)	46.3 (0.83)	42.9 (0.77)	0.004
Positive Affect (PANAS)	34.3 (1.31)	29.5 (1.21)	0.010
Anhedonia (SHAPS)	21.3 (0.87)	24.2 (0.81)	0.017
Opioid Craving (VAS)	11.4 (3.82)	22.2 (3.60)	0.043

MORE vs. SG treatment effects are expressed as estimated marginal means (standard error) from linear mixed effects repeated measures ANCOVA models (post-treatment, follow-up) adjusted for pre-treatment levels of the dependent variable. APNIS = Attention to Positive and Negative Information Scale; PANAS = Positive and Negative Affect Schedule-Short Form; SHAPS = Snaith Hamilton Pleasure and Anhedonia Scale; VAS = opioid craving visual analog scale (0-100mm).

© 2025 Garland EL et al. *JAMA Psychiatry*.

eReferences.

1. Han B, Jones CM, Einstein EB, Dowell D, Compton WM. Prescription Opioid Use Disorder Among Adults Reporting Prescription Opioid Use With or Without Misuse in the United States. *J Clin Psychiatry*. 2024;85(3):56054. doi:10.4088/JCP.24m15258

2. Jackson DC, Malmstadt JR, Larson CL, Davidson RJ. Suppression and enhancement of emotional responses to unpleasant pictures. *Psychophysiology*. 2000;37(04):515-522.

3. Ochsner KN, Bunge SA, Gross JJ, Gabrieli JDE. Rethinking feelings: An fMRI study of the cognitive regulation of emotion. *J Cogn Neurosci*. 2002;14(8):1215-1229.

4. Garland EL, Bryan CJ, Nakamura Y, Froeliger B, Howard MO. Deficits in autonomic indices of emotion regulation and reward processing associated with prescription opioid use and misuse. *Psychopharmacology (Berl)*. 2017;234(4):621-629.

5. Garland, E.L. *Mindfulness-Oriented Recovery Enhancement: An Evidence-Based Treatment for Chronic Pain and Opioid Use*. Guilford Press; 2024.

6. Froeliger B, Mathew AR, McConnell PA, et al. Restructuring reward mechanisms in nicotine addiction: A pilot fMRI study of Mindfulness-Oriented Recovery Enhancement for cigarette smokers. *Evid Based Complement Alternat Med*. 2017;2017:e7018014. doi:10.1155/2017/7018014

7. *MATLAB Version 9.3.0.713579 (R2017b)*. The Mathworks, Inc.; 2017.

8. Delorme A, Makeig S. EEGLAB: an open source toolbox for analysis of single-trial EEG dynamics including independent component analysis. *J Neurosci Methods*. 2004;134(1):9-21. doi:10.1016/j.jneumeth.2003.10.009

9. Bernat EM, Williams WJ, Gehring WJ. Decomposing ERP time–frequency energy using PCA. *Clin Neurophysiol*. 2005;116(6):1314-1334.

10. Thiruchselvam R, Blechert J, Sheppes G, Rydstrom A, Gross JJ. The temporal dynamics of emotion regulation: An EEG study of distraction and reappraisal. *Biol Psychol*. 2011;87(1):84-92.

11. Schupp HT, Cuthbert BN, Bradley MM, Cacioppo JT, Ito T, Lang PJ. Affective picture processing: the late positive potential is modulated by motivational relevance. *Psychophysiology*. 2000;37(2):257-261.

12. Foti D, Hajcak G. Deconstructing Reappraisal: Descriptions Preceding Arousing Pictures Modulate the Subsequent Neural Response. *J Cogn Neurosci*. 2008;20(6):977-988. doi:10.1162/jocn.2008.20066

13. Garland EL. *Mindfulness-Oriented Recovery Enhancement: An Evidence-Based Treatment for Chronic Pain and Opioid Use*. Guilford Press; 2024.

14. Scott JC, Cooke JE, Stanski DR. Electroencephalographic quantitation of opioid effect: comparative pharmacodynamics of fentanyl and sufentanil. *Anesthesiology*. 1991;74(1):34-42.

© 2025 Garland EL et al. *JAMA Psychiatry*.

15. Polunina AG, Davydov DM. EEG spectral power and mean frequencies in early heroin abstinence. *Prog Neuropsychopharmacol Biol Psychiatry*. 2004;28(1):73-82.

16. Pinheiro ES dos S, Queirós FC de, Montoya P, et al. Electroencephalographic Patterns in Chronic Pain: A Systematic Review of the Literature. *PLOS ONE*. 2016;11(2):e0149085. doi:10.1371/journal.pone.0149085

17. Preacher KJ, Hayes AF. Asymptotic and resampling strategies for assessing and comparing indirect effects in multiple mediator models. *Behav Res Methods*. 2008;40(3):879-891.

18. Felder JN, Smoski MJ, Kozink RV, et al. Neural mechanisms of subclinical depressive symptoms in women: a pilot functional brain imaging study. *BMC Psychiatry*. 2012;12(1):152.

19. Frison L, Pocock SJ. Repeated measures in clinical trials: analysis using mean summary statistics and its implications for design. *Stat Med*. 1992;11(13):1685-1704.

© 2025 Garland EL et al. *JAMA Psychiatry*.
