# Supplementary material for: Positive Emotion Dysregulation in Opioid Use Disorder and Normalization by Mindfulness-Oriented Recovery Enhancement: A Secondary Analysis of a Randomized Clinical Trial
Source: JAMA Psychiatry. 2025 Apr 30;82(7):654–62. doi: 10.1001/jamapsychiatry.2025.0569 (PMC12044540; doi:10.1001/jamapsychiatry.2025.0569)
Supplement: Supplement 3. — Data Sharing Statement [file jamapsychiatry-e250569-s003.pdf]

Data Sharing Statement

Garland. Positive Emotion Dysregulation in Opioid Use Disorder and Normalization by Mindfulness-Oriented Recovery Enhancement. *JAMA Psychiatry*. Published April 30, 2025. doi:10.1001/jamapsychiatry.2025.0569

Data
Additional Information: NCT02602535
Data available: Yes
Data types: Deidentified participant data
How to access data: Data (i.e., summary scores) will be shared with a signed data access agreement, submitted to egarland@health.ucsd.edu.
When available: With publication

Supporting Documents
Document types: None

Additional Information
Who can access the data: researchers whose proposed use of the data has been approved
Types of analyses: Meta-analysis
Mechanisms of data availability: with a signed data access agreement
